# Increased expression of placental growth factor in high-grade endometrial carcinoma

**DOI:** 10.3892/or.2012.2178

**Published:** 2012-12-10

**Authors:** LIEVE COENEGRACHTS, STEFANIE SCHRAUWEN, RITA VAN BREE, EVELYN DESPIERRE, CATHERINE LUYTEN, BART JONCKX, JEAN MARIE STASSEN, IGNACE VERGOTE, FRÉDÉRIC AMANT

**Affiliations:** 1 Division of Gynecologic Oncology, Department of Oncology, KU Leuven, B-3000 Leuven; 2 Division of Gynecologic Oncology, Department of Obstetrics and Gynecology, University Hospitals Gasthuisberg, KU Leuven, B-3000 Leuven; 3 Leuven Cancer Institute, University Hospitals Gasthuisberg, KU Leuven, B-3000 Leuven; 4 ThromboGenics NV, B-3001 Heverlee, Belgium

**Keywords:** placental growth factor, angiogenic factor, endometrial cancer

## Abstract

Placental growth factor (PlGF), a homolog of vascular endothelial growth factor (VEGF), exerts pleiotropic functions in cancer by affecting tumor cells as well as endothelial and inflammatory cells. Moreover, PlGF expression correlates with tumor stage, recurrence, metastasis and patient outcome in different types of cancer. Recently, administration of anti-PlGF therapy reduced tumor growth and metastasis in preclinical tumor models. In the present study, we evaluated the diagnostic and prognostic value of systemic and local expression of PlGF in primary endometrial carcinomas. PlGF levels in tumor lysates (n=128) and serum (n=88) of patients with primary endometrial cancer were determined using ELISA. *PlGF* mRNA expression in endometrial carcinoma tissues was quantified by quantitative qRT-PCR. Results were compared to endometrial cancer stage and grade. Systemic PlGF levels were not altered in patients with endometrial cancer (FIGO stage I-II-III) as compared to healthy controls. Only in FIGO stage IV patients, serum PlGF levels were slightly increased. Local PlGF mRNA and protein expression in endometrial tumors progressively increased with tumor grade. In endometrioid carcinomas, *PlGF* mRNA expression was significantly increased in endometrioid grade 3 tumors as compared to normal endometrial tissue. PlGF protein expression was significantly increased in endometrioid grade 2 and 3 carcinomas and in serous carcinomas as compared to normal endometrial tissue. Our study showed that systemic/serum PlGF levels cannot be used as a diagnostic or prognostic marker in endometrial cancer. However, the increased local expression of PlGF, primarily in high-grade carcinomas, underscores the possibility for preclinical assessment of anti-PlGF therapy in endometrial cancer.

## Introduction

Endometrial cancer is the most frequent malignancy of the female genital tract and the fourth most common cancer in women in industrialized countries (1). The increased availability of clinicopathological and molecular data on endometrial carcinomas has led to a dualistic model of endometrial carcinogenesis (2). Type I endometrial carcinomas are endometrioid adenocarcinomas and comprise 80% of all endometrial cancers. These tumors are primarily hormone-sensitive and have a favorable prognosis. By contrast, type II cancers are non-endometrioid carcinomas encompassing mainly serous and clear cell carcinomas (3). Although 75% of endometrial cancers are diagnosed at an early stage with presumed localized disease, approximately 15–20% of these tumors recur. Prognosis of endometrial cancer is related to several factors, including stage, type and histopathological subtype. Moreover, angiogenic factors may also have a prognostic role in patients with gynecological cancer and should therefore be investigated as clinically useful tumor markers (4–7).

Angiogenesis is an essential component of tumor development and is tightly regulated by the interplay between pro- and anti-angiogenic factors. Vascular endothelial growth factor (VEGF) has been established as a potent inducer of tumor angiogenesis through its pro-survival, mitogenic, chemotactic and permeability-enhancing effects on endothelial cells. These effects are mediated by two high-affinity transmembrane tyrosine kinase receptors, VEGF receptor (VEGFR)-1 and VEGFR-2 (8). Placental growth factor (PlGF) is a homolog of VEGF, but selectively signals through VEGFR-1, that is expressed by various cell types including endothelial cells, mural cells, macrophages, bone marrow progenitors and tumor cells (9). In contrast to VEGF, PlGF is redundant during development and homeostasis, but functions at the angiogenic and inflammatory switch in several diseases, including cancer. PlGF exerts herein pleiotropic activities by stimulating angiogenesis, chemo-attracting macrophages and myeloid bone marrow progenitors and inducing the growth, survival and migration of tumor cells and affecting the metastatic niche (10,11). Recently, genetic and pharmacological blockade of PlGF was shown to inhibit tumor growth and metastasis in several preclinical tumor models (11–13).

Given the above-mentioned pleiotropic actions in cancer, the prognostic role of PlGF has been investigated in several cancer types. In various malignancies, including breast and colorectal cancer, the levels of PlGF in plasma, serum and tumors correlated with tumor stage, recurrence and poor survival (14–16). However, for endometrial cancer, the prognostic function of PlGF has yet to be investigated. Since previous studies identified VEGF as an independent prognostic marker in endometrial cancer (4,17) and other studies showed a positive correlation between PlGF levels and disease progression and survival in breast and colorectal cancer (14–16), we hypothesized that PlGF might act as a prognostic factor in endometrial cancer as well. To this end, herein we evaluated both systemic and local PlGF expression in endometrial cancer patients and correlated these levels with histological and clinical patient data. We observed that local expression of PlGF is increased in high grade endometrial carcinomas.

## Materials and methods

### Patient characteristics and collection of tumor tissue and serum samples

In a prospective study at the University Hospitals UZ Leuven, serum samples were collected between November 2008 and March 2010. Patients with initial diagnosis of endometrioid or serous endometrial cancer were included. Serum samples were collected before any form of surgery and/or treatment was initiated. Control blood samples were obtained from healthy women that were examined for routine (non-oncologic) control at the Department of Obstetrics and Gynecology, UZ Leuven.

Fresh frozen tissues were collected and archived from patients undergoing surgery at the Department of Gynecologic Oncology, UZ Leuven, between November 1999 and July 2010. All samples were collected from patients undergoing primary surgeries. Follow-up data (recurrence and survival) were collected for all patients in November 2011. Furthermore, samples of normal endometrium were collected from age-matched patients undergoing surgery for benign pathologies. All pathology specimens were reviewed by the same pathologist to determine histological subtype and differentiation grade. Moreover, from each frozen tumor block that was used for mRNA and protein extraction, a corresponding H&E-stained frozen section was prepared to evaluate the percentage of tumor tissue in the particular sample. Only samples consisting of >70% tumor tissue were included.

This study and the collection of all human samples were approved by the Institutional Review Board of the University Hospitals Leuven and written informed consent was obtained from all patients.

### Quantification of serum PlGF levels by ELISA

Serum PlGF levels were quantified by a Quantikine ELISA according to the manufacturer’s instructions (R&D Systems, Minneapolis, MN, USA).

### RNA extraction and qRT-PCR on endometrial carcinomas

Total RNA was extracted from 100 mg of homogenized tumor tissue using TriPure Reagent (Roche, Mannheim, Germany) followed by phenol/chloroform purification. cDNA was synthesized from equal amounts of RNA using Multiscribe Reverse TaqMan Transcriptase (Life Technologies, Belgium). Quantitative RT-PCR analysis was performed on an ABI 7000 Sequence Detector (Life Technologies) according to the manufacturer’s instructions. Gene expression of *PlGF* was analyzed using a TaqMan Gene Expression assay (ID: HS00182176_m1, Life Technologies) and was normalized for *β-glucoronidase (GUSB)* expression (TaqMan Gene expression assay, human GUSB endogenous control, Life Technologies). Validation experiments showed stable expression of *GUSB*, and equal PCR efficiency for reference and target genes. For quantification of gene expression, relative quantification was performed using the comparative cycle threshold (Ct) method (ΔΔCt method).

### Protein extraction of endometrial carcinoma tissue and ELISA for quantification of PlGF

Protein lysates from endometrial carcinoma samples were prepared using 100 mg of tumor tissue. Tumor tissue was lysed in mammalian cell lysis buffer (50 mM Tris-HCl, 250 mM NaCl, 0.1% SDS, 0.5% deoxycholic acid, 1% Igepal and 1% protease inhibitor cocktail; Sigma-Aldrich) and homogenization was performed in FastPrep Lysing Matrix tubes in a FastPrep-24 Homogenizer instrument (both from MP Biomedicals, Belgium). Protein lysates were stored at −80°C for further analysis. Total protein content was determined using the BCA Protein Assay kit (Thermo Scientific, Pierce). PlGF levels in protein lysates were quantified using a Quantikine ELISA according to the manufacturer’s instructions (R&D Systems). PlGF levels in the tumor lysates were corrected for total protein content in the sample.

### Statistical analysis

Data are represented in Tukey box-and-whisker plots and statistically analyzed using GraphPad Prism 5 software. Statistical significance was analyzed using a Mann-Whitney test for comparison between 2 groups and a one-way ANOVA followed by a Tukey-Kramer multiple comparison test for comparison between multiple groups and multiple testing correction. Univariate analysis of disease-free survival and cancer-specific overall survival was carried out using the Kaplan-Meier method and differences were estimated by the Mantel-Cox (log-rank) test. Multivariate analysis was performed in SPSS. Differences were considered statistically significant at P<0.05.

## Results

### Serum PlGF levels are only increased in late-stage disease endometrial cancer patients

**Figure 1:**
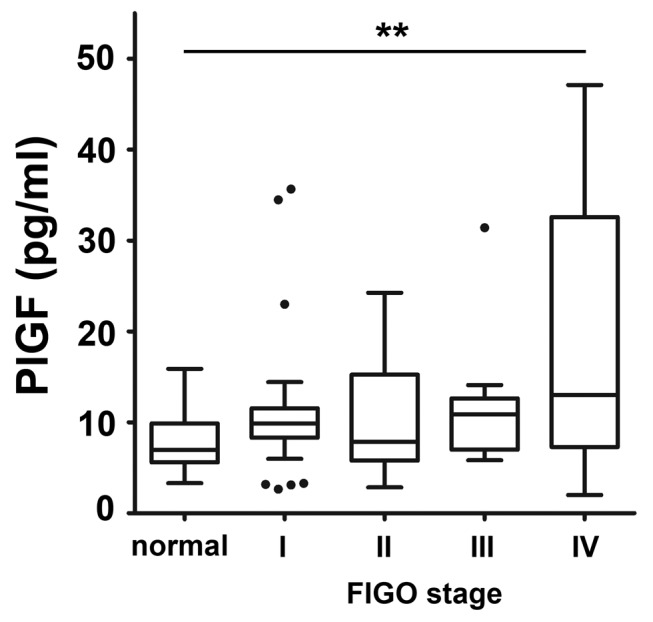
Serum PlGF concentrations in healthy controls and endometrial cancer patients (according to FIGO stage). Horizontal bars represent median values. ^**^P<0.01 vs. normal using one-way ANOVA followed by a Tukey-Kramer multiple comparison test; n=10–37.

In the first part of this study, we aimed to evaluate the prognostic and diagnostic value of systemic PlGF levels in patients with endometrial cancer. To this end, PlGF serum levels were quantified in endometrial cancer patients with different subtypes and at different stages of the disease, FIGO 2009 (18); and these levels were compared to those in healthy control subjects. Patient characteristics are summarized in Table I. Fifty-four patients with endometrioid endometrial cancer (22 with grade 1, 13 with grade 2 and 19 with grade 3), 18 patients with serous endometrial cancer and 16 age-matched healthy-control patients were included (Table I). Thirty-seven patients were diagnosed with FIGO stage I, 10 patients with FIGO stage II, 10 patients with FIGO stage III and 15 patients with FIGO stage IV. PlGF serum levels were significantly different between controls and patients with different FIGO stage (one-way ANOVA: P=0.0088; F-ratio=3.63). Next, the Tukey-Kramer multiple comparison test indicated that no significant differences were observed between serum PlGF levels in endometrial cancer patients with stage I (11.05±1.09 pg/ml), stage II (10.52±2.06 pg/ml) or stage III (11.91±2.34 pg/ml) disease as compared to healthy control subjects (8.11±0.74 pg/ml) (Fig. 1). However, in patients with FIGO stage IV endometrial cancer, PlGF serum levels were significantly increased (18.67±3.86 pg/ml) as compared to healthy subjects and patients with stage I disease (P<0.01 and P<0.05 respectively; Tukey-Kramer multiple comparison test) (Fig. 1). When serum PlGF levels were stratified according to histological subtype, no differences between control subjects, endometrioid or serous cancer patients were observed (data not shown). Our data here indicate that systemic PlGF expression has no diagnostic or prognostic value in endometrial cancer, although we could observe a significant increase of serum PlGF levels in stage IV endometrial cancer patients.

### Local tumoral PlGF expression is increased in high-grade endometrial carcinomas

Subsequently, we analyzed whether local PlGF expression in endometrial carcinomas was increased compared to normal healthy endometrial tissue. Characteristics of the patients included in this study are summarized in Table II. Primary tumor tissue of 96 patients with endometrioid carcinoma (n=39 grade 1, n=27 grade 2, n=30 grade 3) and 23 patients with serous carcinoma was collected and analyzed for PlGF mRNA and protein expression. Also, normal endometrial tissue from 9 women was included. Follow-up data for all patients were collected. After a mean follow-up of 42.7 months (range 1–113), recurrence was noted in 20 patients (15.6%), 8 of which were local (vaginal) and 12 were systemic. Twenty patients (15.6%) succumbed to the disease.

**Figure 2:**
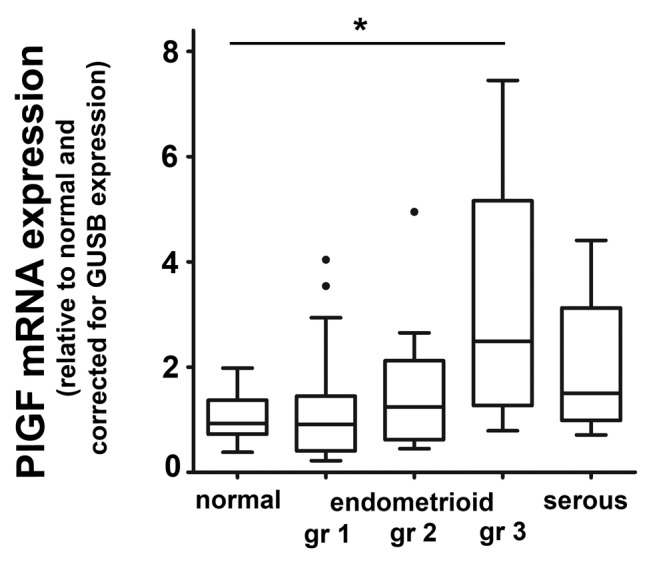
*PlGF* mRNA expression in normal endometrial tissue, endometrioid and serous carcinomas. *PlGF* mRNA expression, corrected for expression of the housekeeping gene *GUSB*, was quantified in endometrial carcinoma samples and normal endometrial tissue samples. All values are expressed relative to the expression levels in normal endometrial tissue. Horizontal bars represent median values. ^*^P<0.05 vs. normal using one-way ANOVA followed by a Tukey-Kramer multiple comparison test; n=9–30.

**Figure 3:**
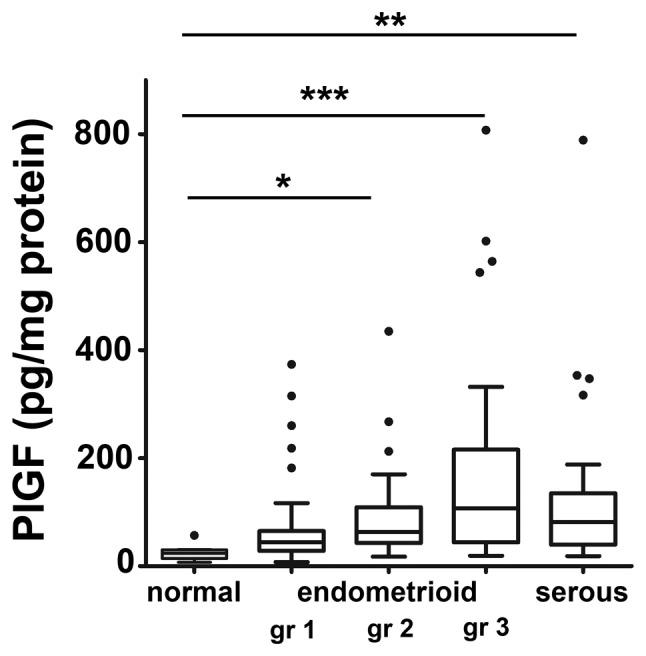
PlGF protein expression in normal endometrial tissue, endometrioid and serous carcinomas. PlGF protein expression levels were quantified by ELISA and corrected for total protein content. Horizontal bars represent median values. ^*^P<0.05, ^**^P<0.01, ^***^P<0.001 vs. normal using one-way ANOVA followed by a Tukey-Kramer multiple comparison test; n=9–30.

First, *PlGF* mRNA expression, normalized for expression of the housekeeping gene *GUSB*, was quantified in tumor tissues and compared to the expression levels in normal endometrial tissue. In endometrioid carcinomas, mean *PlGF* mRNA expression progressively increased with grade (16, 50 and 219% in grade 1, 2 and 3 tumors, respectively), although only significant increases were observed between grade 3 tumors and normal healthy tissue (P<0.05; Fig. 2). No significant differences were observed between *PlGF* mRNA levels in serous carcinomas as compared to normal healthy tissue. Since these data provided a weak correlation between tumor grade and *PlGF* mRNA expression levels, we next quantified PlGF protein levels in tumor lysates of the same tumor specimen. PlGF protein levels in tumor tissues were considerably higher than in healthy non-tumor tissues (118.7±13.8 vs. 25.1±5.4 pg/mg total protein; P<0.001). In particular, PlGF protein expression was significantly increased in grade 2 endometrioid (P<0.01), grade 3 endometrioid (P<0.001) and serous (P<0.01) carcinoma samples as compared to healthy non-tumor tissues (Fig. 3). Although statistical significance differed between protein and mRNA expression, PlGF protein expression correlated well with *PlGF* mRNA expression from the same tissue (r=0.4998; P<0.001; Spearman’s correlation).

### Prognostic significance of PlGF levels in endometrial cancer patients

**Figure 4:**
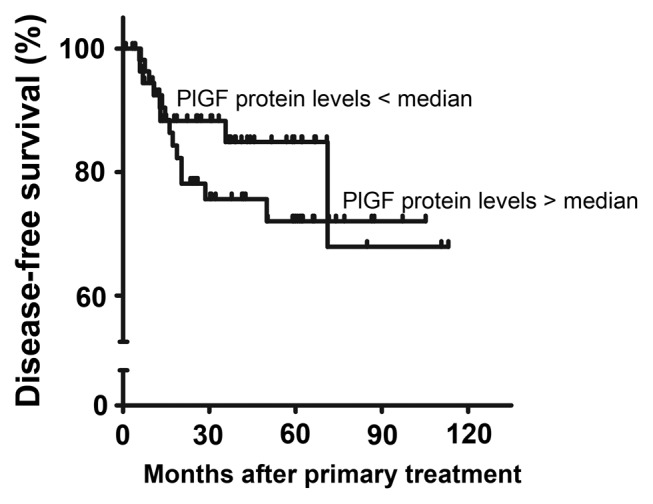
Kaplan-Meier survival curves of disease-free survival for patients with endometrial carcinoma according to low (<median) or high (>median) levels of PlGF protein expression.

**Figure 5:**
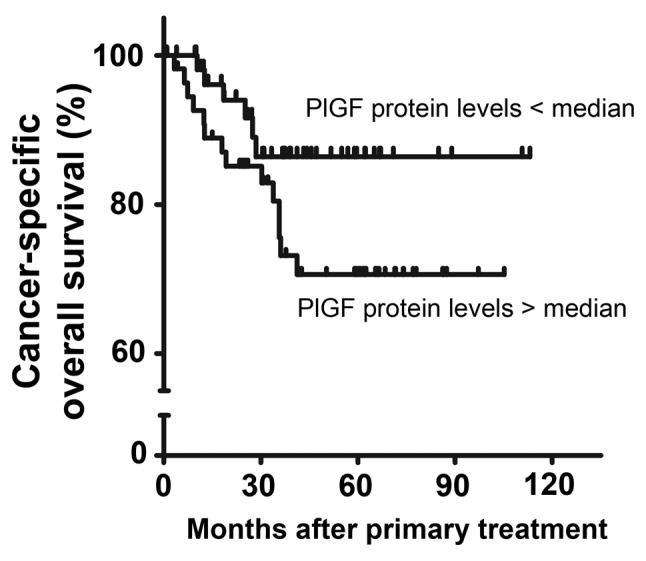
Kaplan-Meier survival curves of cancer-specific overall survival for patients with endometrial carcinoma according to low (<median) or high (>median) levels of PlGF protein expression.

To evaluate whether PlGF levels correlate with clinical outcome of patients with endometrial cancer, univariate and multivariate analyses were performed. Patients were divided into low and high expression groups, based on whether their serum/tumor PlGF protein levels were above or below the median PlGF protein value. For serum PlGF levels, no significant differences in relapse-free survival or overall survival between patients with high or low PlGF serum levels were observed in univariate analysis or in multivariate analyses (data not shown). Also, univariate and multivariate analysis of disease-free survival and cancer-specific overall survival (Figs. 4 and 5) showed no significant differences between patients with high or low local PlGF protein levels in their primary tumor. These data, therefore, indicated that no independent significance was achieved for the PlGF signature compared with standard clinicopathological factors such as tumor grade and FIGO stage.

## Discussion

Numerous angiogenic factors are currently considered and further investigated as potential therapeutic targets. PlGF, a homolog of VEGF, is known to be redundant during development and homeostasis, but is crucial in regulating the angiogenic switch in pathological conditions (10,19). In particular, cumulating evidence points to a pleiotropic role of PlGF in promoting human cancer progression, therefore making PlGF an attractive therapeutic candidate (9). To date, no data on PlGF’s expression pattern in gynecological cancers, and, in particular, endometrial cancer, are available. Our study is the first to quantify PlGF expression levels, both systemic and local, in endometrial cancer and to correlate these expression levels with the available clinicopathological parameters.

We first investigated the prognostic and diagnostic value of PlGF in endometrial cancer. In contrast to VEGF, of which the serum levels are increased in patients with endometrial cancer (20), we did not observe any differences in serum PlGF levels between patients with endometrial cancer and age-matched healthy controls. Our data, therefore, indicate that circulating PlGF cannot be used as a diagnostic or prognostic marker in endometrial cancer. These observations are in line with previous studies since the prognostic impact of circulating PlGF has been controversial in different types of tumors (21–23). Nevertheless, we did observe a significant increase in serum PlGF levels in patients with late-stage (FIGO stage IV) disease, an observation that may be due to the general spread of the disease since we also showed that PlGF is locally produced by endometrial carcinomas.

Whereas the prognostic value of circulating PlGF in cancer is controversial and mostly unclear, studies on local tumor expression of PlGF and its correlation with clinicopathological parameters are more precise. PlGF expression is increased in several different types of tumor, including mesothelioma (22), breast (14,15), non-small cell lung (24), colorectal (16), and gastric carcinomas (25). Moreover, elevated PlGF levels in these tumors were associated with increased risk for recurrence, metastasis and reduced survival. In the present study we provided evidence that PlGF expression is also increased in endometrial carcinomas as compared to normal endometrial tissue. Moreover, the expression of PlGF protein was markedly higher in more aggressive tumor subtypes (endometrioid grade 3 and serous), an observation that was previously described for breast cancer as well (15). Our data also support the binary classification system for endometrial cancer into type I and II tumors. The precise allocation of endometrial tumors according to this dualistic model has been highly debated, since, based on clinical findings, grade 3 endometrioid carcinomas have been classified as type II by some groups (3,26). Our observations here confirm this classification since PlGF expression levels were mostly increased in grade 3 endometrioid and serous carcinomas, suggesting that these tumors form a separate subgroup of more aggressive tumors.

In several types of tumor, a positive correlation between high PlGF expression levels and reduced prognosis has previously been shown. However, in our univariate and multivariate analyses, there was no correlation between high PlGF levels and reduced disease-free survival or cancer-specific overall survival.

To evaluate PlGF expression in endometrial carcinomas, we quantified both mRNA and protein expression. Although we demonstrated that PlGF protein expression was significantly increased in high-grade endometrial carcinomas, *PlGF* mRNA expression failed to reach statistical significance in high-grade tumors. This discrepancy between mRNA and protein expression of PlGF can be caused by posttranscriptional regulation and differences in mRNA and protein turnover rates. Nevertheless, a positive correlation between PlGF mRNA and protein levels was present in our data.

We showed that PlGF levels are markedly increased in endometrial carcinomas, primarily in high-grade tumors, suggesting that PlGF might act as an important local growth factor to stimulate tumor growth. Since pre-clinical studies have proven the benefit of PlGF-targeting antibodies (11–13) and clinical studies have already shown the safety of these agents (27), the use of PlGF-targeting strategies in endometrial cancer, whether or not in combination with other treatment options, may be considered in the future.

## Figures and Tables

**Table I:** Characteristics of patients included in the serum analysis study.

Patient characteristics	N	%
Total number of patients	88	
Age (years)		
Mean	63.3	
Range	36–93	
Histological subtype		
Normal endometrium	16	18.2
Endometrioid	54	61.4
Serous	18	20.4
FIGO stage (2009)		
Healthy control	16	18.2
I	37	42.0
II	10	11.4
III	10	11.4
IV	15	17.0
Differentiation grade		
Normal endometrium	16	18.2
Low (grade 1)	22	25.0
Intermediate (grade 2)	13	14.8
High (grade 3)	37	42.0

**Table II:** Characteristics of patients included in the study for local expression of PlGF.

Patient characteristics	N	%
Total number of patients	128	
Age (years)		
Mean	66.2	
Range	34–93	
Histological subtype		
Normal endometrium	9	7.0
Endometrioid	96	75.0
Serous	23	18.0
FIGO stage (2009)		
Healthy control	9	7.0
I	68	53.1
II	15	11.7
III	20	15.7
IV	16	12.5
Differentiation grade		
Normal endometrium	9	7.0
Low (grade 1)	39	30.5
Intermediate (grade 2)	27	21.1
High (grade 3)	53	41.4
